# Coded Permutation Entropy: A Measure for Dynamical Changes Based on the Secondary Partitioning of Amplitude Information

**DOI:** 10.3390/e22020187

**Published:** 2020-02-06

**Authors:** Huan Kang, Xiaofeng Zhang, Guangbin Zhang

**Affiliations:** School of Physics and Information Technology, Shaanxi Normal University, Xi’an 710119, China; kanghuan@snnu.edu.cn (H.K.); guangbinzhang@snnu.edu.cn (G.Z.)

**Keywords:** permutation entropy, coded sequence matrix, dynamical change detection, time series

## Abstract

An improved permutation entropy (PE) algorithm named coded permutation entropy (CPE) is proposed in this paper to optimize the problems existing in PE based on the secondary partitioning. The principle of CPE algorithm is given, and the performance of it for dynamical change detection is analyzed using synthetic signal, logistic map and Lorenz map. The detection ability of CPE algorithm in different signal-to-noise ratios (SNR) is studied and the algorithm complexity is discussed. The results show that CPE can accurately capture minor feature information and amplify the detection results of dynamical changes compared with PE, weighted permutation entropy (WPE) and amplitude-aware permutation entropy (AAPE), but it has less robustness to noise and requires a higher computation cost than the others. Finally, we use the new algorithm to analyze the rolling bearing fault signals. The application of actual signals illustrates that CPE performs better in detecting abnormal pulse of the rolling bearing when the embedded dimension is small. From all the analyses in this paper, we find that CPE has a better performance for dynamical change detection compared with the other three algorithms when there is a larger repetition rate of permutation pattern in the position sequences.

## 1. Introduction

Detecting the dynamical changes of complex systems and distinguishing the complexity of output time series offer great practical significance in physics, biomedicine, engineering and economics. Various information-theoretic methods have been developed to measure complexity, such as entropy analysis [1,2,3,4,5,6,7], Lyapunov exponents [8,9], statistical complexity measure [10,11], correlation dimension [12], and symbolic dynamics [13]. Among these methods, entropy analysis is widely used in many fields because its principle is simple, and it can reflect the characteristics of the system effectively.

In 2002, Bandt and Pompe proposed permutation entropy (PE) to measure the natural complexity of time series [4]. After that, this algorithm has attracted the attention of many researchers because it has superior robustness and requires less computation [14,15,16]. Many derivative algorithms, such as multiscale permutation entropy [17,18], weighted permutation entropy (WPE) [19,20,21], Rényi permutation entropy [22,23], generalized permutation entropy [24], multivariate permutation entropy [25], and amplitude-aware permutation entropy (AAPE) [26] have been proposed to extend the application of PE to a variety of fields.

PE algorithm is based on the probability distribution of the permutation pattern obtained by calculating the frequency of each permutation pattern in all position sequence. However, it does not mean that the reconstructed component is completely consistent before ascending order, although they have the same positions sequence [19,25,26,27,28]. First, if the position sequence is *‘1,2,3’*, the original data that is indicated by *‘2’* may be located in three different areas [19,25]. Secondly, it may have the same position sequence when a constant value is added to the original data as shown in Reference [26]. Thirdly, it also has the same position sequence when equivalent elements appear in the original data [27,28]. In PE algorithm, all the above situations are marked as the same permutation pattern. Therefore, PE is not accurate when it is used to divide those cases. This is because only counting the frequency of occurrence of each permutation pattern can result in the loss of much detailed information. To solve the first, Fadlallah et al. in 2013 proposed WPE which introduced the amplitude information into PE algorithm using variance to represent the probability [19,20,21]. However, WPE does not solve the problem when a constant value is added to the original data because its variance does not change any more. In 2016, Azami et al. proposed amplitude-aware permutation entropy (AAPE) to settle the second and the third by adding a variable contribution parameter *A*, which is an adjusting coefficient related to the mean value and the difference between consecutive samples to make the algorithm more flexible [26]. The simulation results show that WPE and AAPE greatly improve the detection ability of amplitude changed signal compared to PE. However, dynamical changes do not only exist in terms of amplitude, and system complexity change detection is also very common. Based on this point, it is not necessarily advantageous to use WPE or AAPE in detecting dynamical changes of system complexity compared to PE.

In addition to the above problem, another question for PE in capturing the characteristic information of the data is that the selection of embedding dimension *m* is excruciating. If *m* is small, the reconstructed component will contain few distinct patterns, and it is detrimental to the extraction of abnormal feature [29,30,31]. For example, the potential permutation pattern of m=3 is 3!=6, and the detection result of dynamical changes when m>3 is better than m=3 [4,24]. Considering in this way, using a large value of *m* is fine. However, the larger *m* is, the larger the data length *N* in PE algorithm will be needed. The relationship between *m* and *N* is that *N* should be considerably larger than m! [4,22]. In some applications, the length of data is not always sufficient.

The main reason for the above two problems is that it is too rough to calculate the probability only using the permutation pattern as the partitioning criterion in PE algorithm. In Reference [25], He et al. proposed a concept which has the intention of secondary partitioning, and they applied their idea in the multivariate system and presented multivariate permutation entropy (MvPE). In their concept, each permutation pattern in PE is divided into three sub-patterns according to where the intermediate element is. However, MvPE is used to measure the complexity of multivariate systems for embedding dimension m=3, and some difficulties prevent its application when m>3. In this paper, we make a secondary partitioning, which improving the accuracy of partitioning based on permutation pattern and overcoming the incompatibility of MvPE algorithm for *m*, and propose coded permutation entropy (CPE).

The paper is organized as follows. In Section 2, CPE algorithm is explained. Dynamical change detection and algorithm complexity are presented in Section 3. We analyze the dynamical detection of three rolling bearing fault signals using CPE algorithm in Section 4. The concluding remarks are recorded in Section 5.

## 2. Methodologies

### 2.1. Permutation Entropy

For a scalar sequence {x(i),i=1,2,…,N}, it is embedded into a *m*-dimension state space as:

(1) X(j)=[x(j),x(j+τ),…,x(j+(m−1)τ)],

where j=1,2,…,N−(m−1)τ, *N* is the data length of scalar sequence, *m* and τ denote the embedding dimension and the time delay and m=3,…,7 and τ=1,2,3… [4].

Then, the reconstructed component X(j) is arranged in an increasing order as $x\left(j+\left(k_{1}-1\right)\tau \right)\leq x\left(j+\left(k_{2}-1\right)\tau \right)\leq \ldots \leq x\left(j+\left(k_{m}-1\right)\tau \right)$. When an equality occurs, such as $x\left(j+\left(k_{l1}-1\right)\tau \right)=x\left(j+\left(k_{l2}-1\right)\tau \right)$, *x* will be permuted according to the size of $k_{l1}$ and $k_{l2}$; specifically, if $k_{l1}<k_{l2}$, let $x\left(j+\left(k_{l1}-1\right)\tau \right)\leq x\left(j+\left(k_{l2}-1\right)\tau \right)$. Therefore, the position sequence which corresponds to the permutation type in Reference [4] can be obtained as follow:

(2) $$S\left(j\right)=\left\{k_{1},k_{2},\ldots ,k_{m}\right\},$$

where $k_{l}$ is the position index of $x(j+\left(k_{l}-1\right)\tau )$ in X(j).

For a certain embedding dimension *m*, there are m! potential permutations patterns, and each permutation pattern can be known as F(v),1≤v≤m! [4].

Next, we calculate the frequency of each F(v) in all S(j), and Num(v) is the count of each F(v). Then, the probability can be marked as

(3) $$P\left(v\right)=\frac{Num(v)}{N-(m-1)\tau },1\leq v\leq m!.$$

Finally, PE is computed by

(4) $$H_{PE}\left(m\right)=-\sum_{v=1}^{m!}P\left(v\right)\ln P\left(v\right).$$

It is clear that $0\leq H_{PE}\left(m\right)\leq \ln\left(m!\right)$. $H_{PE}\left(m\right)$ has the maximum value ln(m!) when $P\left(v\right)=\frac{1}{m!}$. Generally, $H_{PE}\left(m\right)$ is normalized by ln(m!) as $h_{PE}\left(m\right)=\frac{H_{PE}\left(m\right)}{\ln(m!)}$.

### 2.2. Coded Permutation Entropy

The reason WPE and AAPE have a sensitive detection ability regarding the changes in amplitude is that they use specific amplitude information directly in probability calculation. Therefore, they cannot show good performance if the change of amplitude in data is very small whereas prominent changes in system complexity are expected. To solve this problem, we use the initial data as a hidden assistant rather than take it directly, and propose an improved algorithm named CPE. The processes of CPE algorithm are as follows:

**Step** **1** The steps before getting the position sequence S(j) for CPE are the same as those for PE.
**Step** **2** Obtain the serial number which is the position index of each S(j) in all permutation pattern F(v), and record them in column 1 of row *j* of matrix *Q*, namely Q(j,1).
**Step** **3** Average the reconstructed components X(j) which have the same position sequence, and call the result as the mean sequence Me(v),1≤v≤m!.
**Step** **4** Compare the size of each element in reconstructed component X(j) with its corresponding mean sequence Me(v) using Equation (5), and record the comparison results in column 1+k of row *j* of matrix *Q*.
  (5) $$\begin{array}{c} Q\left(j,1+k\right)=\left\{\begin{array}{c} 2,X(j,k)>Me(v,k)\\ 1,X(j,k)=Me(v,k)\\ 0,X(j,k)<Me(v,k), \end{array}\right.j=1,2,\ldots ,N-\left(m-1\right)\tau ,k=1,2,\ldots ,m. \end{array}$$
**Step** **5** A coded sequence matrix Q(N−(m−1)τ,1+m) is constructed and each row of it can be named the coded sequence. Obviously, the number of potential patterns existing in CPE is $m!\cdot 3^{m}$, and so the probability of unique coded sequence is calculated as:
  (6) $$P\left(r\right)=\frac{Num(r)}{N-(m-1)\tau },1\leq r\leq m!\cdot 3^{m}.$$
**Step** **6** CPE can be obtained by substituting the probability P(r) into the Shannon entropy formula, namely
  (7) $$H_{CPE}\left(m\right)=-\sum_{r=1}^{m!\cdot 3^{m}}P\left(r\right)\ln P\left(r\right).$$

Similarly, the normalized expression of $H_{CPE}\left(m\right)$ is $h_{CPE}\left(m\right)=\frac{H_{CPE}\left(m\right)}{\ln\left(m!\cdot 3^{m}\right)}$. Since PE and CPE are normalized on different base, we use Equation (4) and Equation (7) to calculate the values of PE and CPE in the next analyses.

**Figure 1:**
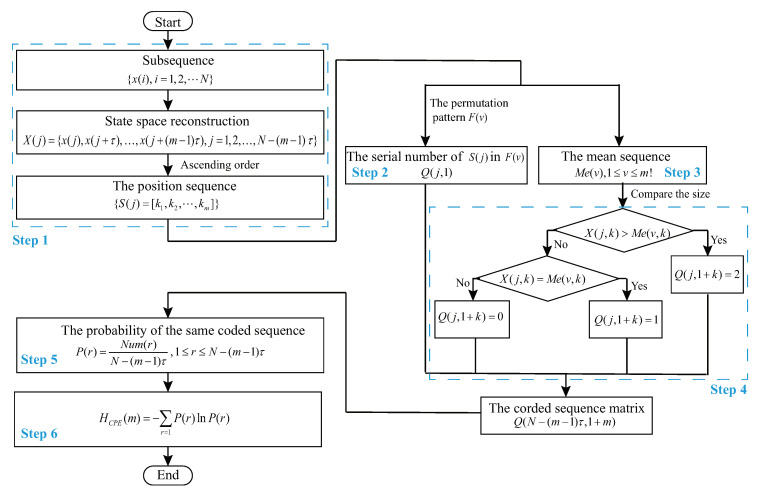
The flow chart of CPE algorithm. The left part of this flow chart is the basic steps, and the right is the key steps of CPE algorithm called the secondary partitioning which aims to obtain the coded sequence matrix Q(N−(m−1)τ,1+m).

**Figure 2:**
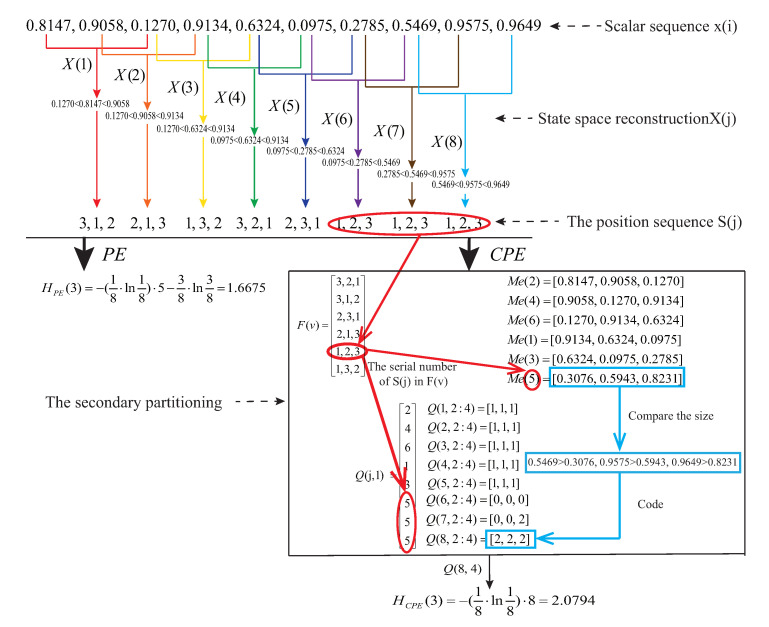
The processes of calculating PE and CPE for a simple example. For the secondary partitioning in CPE, using the serial number constructs Q(j,1) as shown in the bold red line, and Q(j,2:4) records the size of the relationship between X(j) and Me(v) as the bold blue line shown.

Based on the above statements about CPE, the flow chart of CPE algorithm is shown in Figure 1. A simple example is presented to illustrate the calculation processes of PE and CPE as shown in Figure 2. The scalar sequence {x(i)} of this example is {0.8147, 0.9058, 0.1270, 0.9134, 0.6324, 0.0975, 0.2785, 0.5469, 0.9575, 0.9649} which is the random number drawn from the standard normal distribution. In Figure 2, the position sequence *‘1, 2, 3’* is the fifth in the permutation pattern of m=3, and so we set Q(j,1)=5 when j=6,7,8 which is shown in the bold red line of Figure 2. Due to the reconstructed component X(6), X(7) and X(8) have the same position sequence *‘1, 2, 3’*, the corresponding mean sequence can be recorded by calculating the mean of X(6),X(7),X(8), namely Me(5)=[0.3076,0.5943,0.8231]. Then we compare the size between X(6),X(7),X(8) and Me(5) respectively, and get the coded sequences Q(6,2:4), Q(7,2:4) and Q(8,2:4) (we only present the specific process of obtaining Q(8,2:4) as shown in the bold blue line of Figure 2, and Q(8,2:4)=[2,2,2] means the value of column 2 through 4 of row 8 in matrix Q(8,4) is 2, 2 and 2, respectively). It is clear that CPE successfully divides the reconstructed component X(6),X(7),X(8) into three classes by the process of the secondary partitioning which is circled by a black box in Figure 2. In particular, CPE will degenerate to PE when the probability P(r) in Equation (6) is presented by the frequency of the same serial number in Q(j,1) according to Figure 1 and Figure 2. The same procedures can be used to achieve the secondary partitioning for m>3.

## 3. Simulation Analyses

The number of potential permutation pattern in PE, WPE and AAPE is m!, and the pattern in CPE is $m!\cdot 3^{m}$ due to the introduction of the secondary partitioning. Because of that, we can expect CPE to be more significant than PE, WPE and AAPE in the measure of dynamical changes. Three models, which are generally used in dynamical change detection, are built to verify the performance of CPE algorithm, namely synthetic signal, discrete standard model and continuous standard model. In addition to presenting the corresponding entropy graph, the standard deviation (Std) which is the most common formula to reflect the discreteness of a set of data is also calculated to show the effect of dynamical change detection from the perspective of quantitative analysis. Moreover, the experiments in the case of noise and the algorithm complexity are also analyzed.

### 3.1. Synthetic Signal

**Figure 3:**
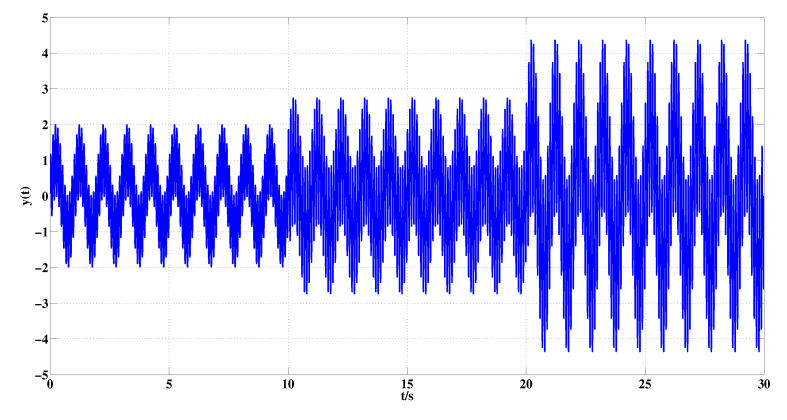
Synthetic signal y(t).

A synthetic signal y(t) is produced using the following equation:

(8) $$\begin{array}{c} y\left(t\right)=\left\{\begin{array}{cc} \sin(2\pi \cdot t)+\sin(2\pi \cdot 10\cdot t) & 0<t\leq 10\\ \sin(2\pi \cdot t)+\sin(2\pi \cdot 10\cdot t)+\sin(2\pi \cdot 20\cdot t) & 10<t\leq 20\\ 2\cdot \sin(2\pi \cdot t)+1.5\cdot \sin(2\pi \cdot 10\cdot t)+1.25\cdot \sin(2\pi \cdot 20\cdot t) & 20<t\leq 30 \end{array}\right. \end{array}$$

where the sampling frequency is 1000 Hz and the sampling time is 30 s. The curve of synthetic signal y(t) is shown in Figure 3.

**Figure 4:**
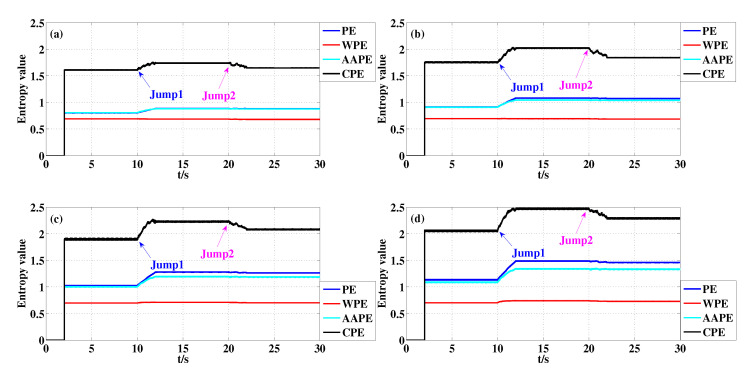
Detecting dynamical changes existing in the synthetic signal y(t) with different *m* using PE, WPE, AAPE, and CPE: (**a**) m=3,τ=1; (**b**) m=4,τ=1; (**c**) m=5,τ=1; (**d**) m=6,τ=1.

Figure 4a–d show the detection results of PE, WPE, AAPE and CPE for the above time series y(t) when m=3,τ=1, m=4,τ=1, m=5,τ=1 and m=6,τ=1, respectively (The value of parameter *A* existing in AAPE algorithm is 0.5 in all experiments in this paper). A 2000-sample sliding window with the increment of 1 is used in this computation. From Figure 4, we see that the entropy values of PE, WPE, AAPE and CPE increase suddenly at t=10 s (Jump1), and then it decrease at t=20 s (Jump2). The reason is that y(t) is more complex between 10 s and 20 s, so the corresponding entropy value is larger than those of 0 s–10 s. The only difference for y(t) between 10 s–20 s and 20 s–30 s is the amplitude which cause the decrease of entropy value at t=20 s. Whatever *m* are, the jumps of CPE are the most obvious among than PE, WPE, and AAPE not only for t=10 s but also for t=20 s. Moreover, the Std and the jumps of entropy value of PE, WPE, AAPE and CPE for synthetic signal y(t) when *m* takes on different values are recorded in Table 1. It is clear to find that the Std and the jumps of CPE are the largest in these four algorithms whatever *m* are. These two aspects indicate that CPE algorithm has some advantages not only for the complexity dynamical change detection but also for the amplitude.

### 3.2. Discrete Standard Model

A transient logistic map can be presented as [29]:

(9) $$x_{i+1}=r\cdot x_{i}\cdot \left(1-x_{i}\right).$$

**Figure 5:**
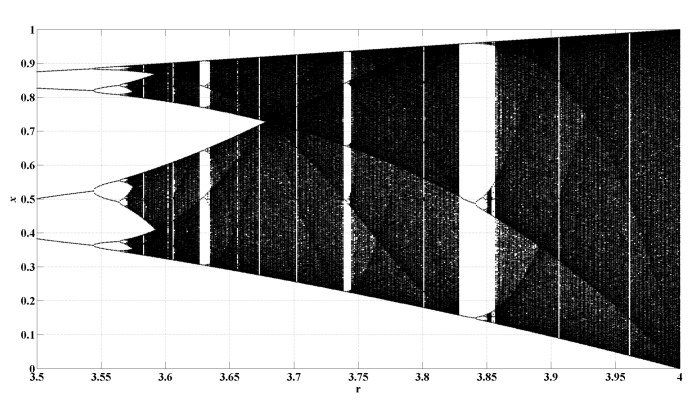
The transient logistic map.

In this example, $x_{0}=0.65$, r(0)=3.5 and *r* is consistently increasing to r=4 by a step of 0.001. For each value of *r*, a time series *x* with a length of 3000 is generated. To discard transients, we only use the last 2000 samples of this time series, as shown in Figure 5 [24].

**Figure 6:**
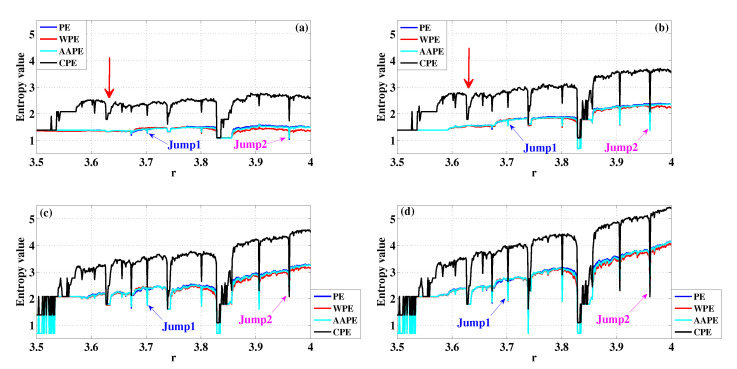
Detecting dynamical changes existing in the transient logistic map with different *m* using PE, WPE, AAPE, and CPE: (**a**) m=3,τ=1; (**b**) m=4,τ=1; (**c**) m=5,τ=1; (**d**) m=6,τ=1.

Figure 6 shows the results of PE, WPE, AAPE, and CPE for the dynamical change detection of the logistic map when *m* are set as different values. From Figure 6, we find that PE, WPE and AAPE have a similar detection result for the chaotic window in the logistic map whatever the value of *m* are, and CPE decreases more than those of other three algorithms in the chaotic window. Although WPE was proposed to consider the amplitude difference of the same position sequences, it does not significantly improve the detection ability of dynamical changes of chaotic windows in the logistic map compared to PE. We also observe that PE, WPE and AAPE have no prominent decrease when r=3.628 whether it is m=3 or m=4. However, there is a significant entropy decrease in CPE, which is marked by a red arrow in Figure 6a,b. Table 2 presents the corresponding Std and the jumps of entropy value for the logistic map when m=3,4,5,6. Consistent with the result of entropy graph, the Std and the jumps of CPE are still the largest in these four algorithms. It can be concluded that CPE amplifies the detection results of dynamical changes in the logistic map, and has a weak dependence on parameter *m* when we hope to observe the dynamical changes accurately.

### 3.3. Continuous Standard Model

In the above simulation, we analyzed the ability of CPE to detect the dynamical changes for a discrete time system. Next, we examine a continuous time system described by the following transient Lorenz equations [29]:

(10) $$\begin{array}{ccc} \frac{dx}{dt} & = & -10(x-y)\\ \frac{dy}{dt} & = & -xz+r(t)x-y\\ \frac{dz}{dt} & = & xy-\frac{8}{3}z. \end{array}$$

**Figure 7:**
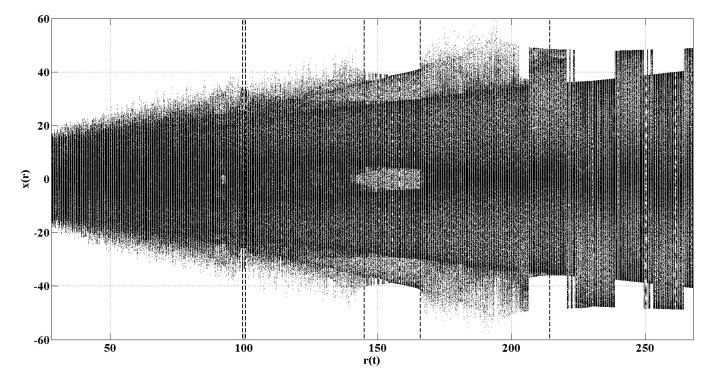
The transient Lorenz map.

The system is solved using a fourth-order Runge–Kutta method with a time step Δt=0.01. In our simulation, x(0)=−1, y(0)=0, z(0)=−1, r(0)=28 and r(t) increases to r(t)=268 by a step of 0.2. For each r(t), we obtain a series with a length of 3000. It is consistent with the logistic map that we only take the last 2000 data to calculate the entropy, and the transient Lorenz map is shown in Figure 7. Theoretically, there are three periodic windows, which existing in 99.524<r(t)<100.795, 145<r(t)<166 and r(t)>214.4, as indicated by dashed vertical lines in Figure 7 [32].

**Figure 8:**
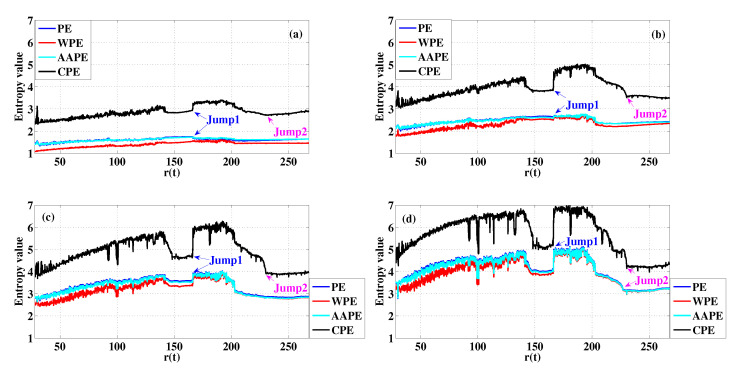
Detecting dynamical changes existing in the transient Lorenz map with different *m* using PE, WPE, AAPE, and CPE: (**a**) m=3,τ=10; (**b**) m=4,τ=10; (**c**) m=5,τ=10; (**d**) m=6,τ=10.

The choices of both *m* and τ are related to the time step Δt. Based on this point, we set m=3, τ=10, m=4,τ=10, m=5,τ=10 and m=6,τ=10 [32], and the corresponding entropy value curves are presented in Figure 8a–d, respectively. It is consistent with the logistic map that PE, WPE and AAPE have similar detection results when a periodic window appears in the transient Lorenz map. According to Figure 8, we observe that all these curves of CPE capture the periodic windows at r(n)=166 (Jump1) very well whatever m=3 or m=4. The change existing in r(n)=230.4 (Jump2) also can be detected using CPE when m=4 rather than m=6 for PE, WPE and AAPE. It is very beneficial for dynamical change detection if we can produce good results when *m* is small. Furthermore, the corresponding Std and the jumps of CPE also reflect these performances in complexity dynamical change detection, which is recorded in Table 3.

For PE, Bandt and Pompe recommend m=3,…,7 [4]. However, the number of F(v) in m=3 and m=4 is 6 and 24, hence, it is not very large, which is the reason that m=3,4 is not the best choice for PE algorithm to detect the dynamical changes [29]. WPE takes into account the amplitude differences between the same position sequences, and it improve the performance of PE for the detection of abnormal amplitude change [19]. However, for the above dynamical change detection in system complexity, WPE and AAPE do not demonstrate better detection ability than PE. Both for chaotic windows detection of the logistic map and periodic windows detection of the Lorenz map, CPE shows a superior detection result compared to PE, WPE and AAPE. This result illustrates that the secondary partitioning is very useful in extracting the feature information. In particular, it can greatly improve the detection performance of PE when m=3,4.

### 3.4. Robustness to Noise

To study the dynamical change detection performance of these four algorithms at different signal-to-noise ratios (SNR), we add white Gaussian noise to the two series, and the power of them is expressed in dB. The data length of these two series are 20000, and they produced by Equation (9) when set r(n)=3.961 and r(n)=3.962, respectively. In the logistic map, x(n) is a periodic series when r(n)=3.961 and it is a chaotic series when r(n)=3.962.

**Figure 9:**
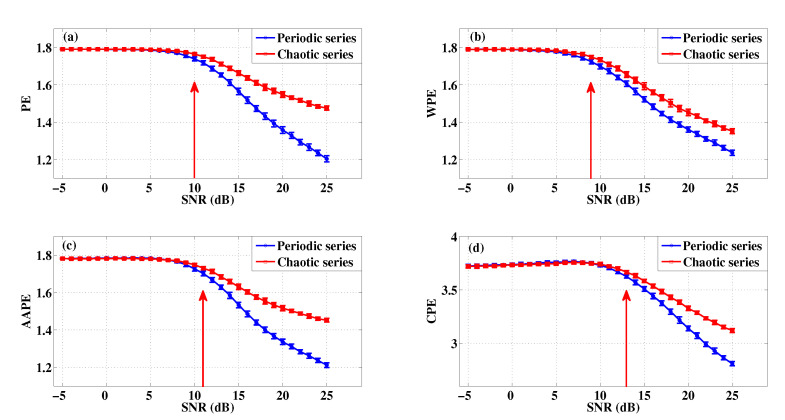
The performance of four algorithms in distinguishing periodic series and chaotic series under different SNR when m=3,τ=1: (**a**) PE; (**b**) WPE; (**c**) AAPE; (**d**) CPE.

The 30 statistical values of PE, WPE, AAPE, and CPE for different SNR when m=3,τ=1 are presented in Figure 9 using errorbar [33]. As we all know, it is certain that the entropy value will decrease with the increase of SNR at step of 1 from SNR = −5. From Figure 9, we find that the entropy value of the periodic series decrease more than the chaotic series with the increasing of SNR from −5 to 25, and it is consistent with the detection result of logistic map without noise as show in Figure 6. Furthermore, either for the periodic series or for the chaotic series, the entropy value of CPE decreases the most from the perspective of absolute values in these four algorithms. As shown in Figure 9, with the increasing of SNR, CPE starts distinguishing these two series when the SNR close to 13, and it is indistinguishable before that. The corresponding SNR for PE, WPE, and AAPE is 10, 9 and 11, respectively. These results show that CPE has less robustness to noise than PE, WPE, and AAPE.

### 3.5. The Complexity of Four Algorithms

The complexity of four algorithms is discussed in this subsection. All the experiments are run in MATLAB R2014a on the same computer, with a CPU model number of i5-7500 and RAM size of 16 GB. The running time (per second) of four algorithms for the above three models when *m* has different values are presented in Table 4. From Table 4, we find that the running time is increasing as the increase of *m* for all these four algorithms. When *m* is constant, the running time of PE algorithm is the shortest for these three signals. For m=3, the running time of WPE is slightly longer than CPE. When m=4,5,6, the running time of the four algorithms is sorted as PE, AAPE, WPE, and CPE from small to large basically. The running time of four algorithms for logistic map when m=6 is an exception. The reason for WPE and CPE require more running time is that WPE adds the step of calculating the weighted value [19], and CPE adds the steps of computing the mean sequence and comparing the size of the corresponding element. Furthermore, we calculate the growth rate (Gr) of running time of CPE referring to PE as shown in Table 5, and ‘Average’ in Table 5 means the average of Gr for three signals under the same *m*. Here, Gr can be got by $Gr=\frac{(T_{CPE}-T_{PE})\cdot 100\%}{T_{PE}}$, where $T_{CPE}$ means the running time of CPE and $T_{PE}$ represents the running time of PE. The Gr of running time of CPE to PE is 121% if we take the mean of ‘Average’ at different *m*. A similar operation can be used to calculate the Gr of CPE referring to WPE and AAPE, and the corresponding mean value of ‘Average’ is 25% and 72% respectively.

The running time of algorithm is closely related to the other factors such as computer configuration. Therefore, we also list the time complexity of four algorithms, which is shown in Table 6. It is evident to see that the time complexity can be sorted as PE, AAPE, WPE and CPE from small to large, which is basically consistent with the conclusion obtained by running time. Therefore, both from the perspective of running time and time complexity of algorithm, the computation cost of CPE is the largest in these four algorithms.

## 4. Apply CPE to Rolling Bearing Fault Detection

Rolling element bearings are one of the most prevalent components in rotating machines, and the failure of the bearing is the most frequent reasons for machine breakdown. To verify the effectiveness of CPE algorithm in actual application, the fault data of rolling bearing from Case Western Reserve University are used [34,35,36], and they are inner race fault (IRF), ball fault (BF) and outer race fault (ORF). The sample frequency of them is 12,000 Hz, and the pulse in these signals are considered to be the dynamical changes of actual data [37]. In this experiment, the samples of 0–0.5 s, 0–10 s and 0–0.5 s in these signals are employed as the test data, the window length *w* is set to 200 samples when analyze IRF and ORF signals and it is equal to 500 for BF signal, and the move step is 1 for these three bearing signals.

**Figure 10:**
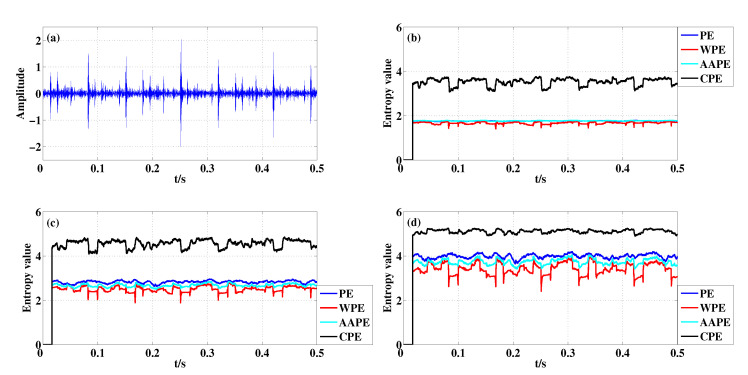
Detecting abnormal pulse existing in IRF signal with different *m* using PE, WPE, AAPE, and CPE: (**a**) The IRF signal; (**b**) m=3,τ=1; (**c**) m=4,τ=1; (**d**) m=5,τ=1.

The IRF signal is plotted in Figure 10a. Figure 10b–d present the corresponding entropy curves for different *m*. From Figure 10, it is obvious that the entropy value has a sudden drop when a pulse appeared and the low value lasts for a window length, and the decrease of CPE is the most significant compared with PE, WPE and AAPE when m=3 and m=4. However, when m=5, CPE has less pulse detection ability than WPE. The reason is that there are few reconstruct components with the same permutation pattern for w=200 when m=5, and so the advantage of CPE are not prominent in this case. From the aspect of quantitative analysis, when m=3 and m=4, the detection result of CPE are more evident than that by PE, WPE and AAPE because the Std of CPE is larger than the others as shown in Table 7.

**Figure 11:**
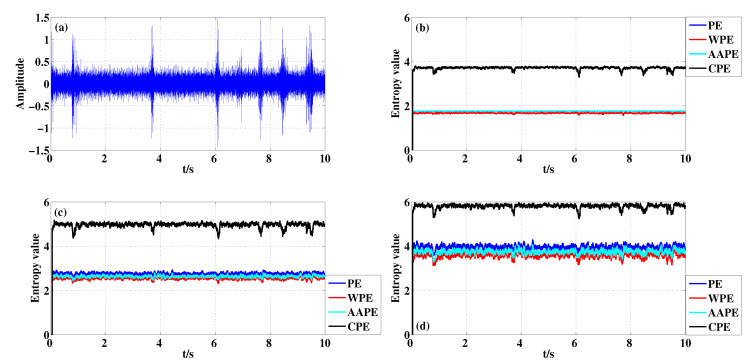
Detecting abnormal pulse existing in BF signal with different *m* using PE, WPE, AAPE, and CPE: (**a**) The BF signal; (**b**) m=3,τ=1; (**c**) m=4,τ=1; (**d**) m=5,τ=1.

For BF signal which is shown in Figure 11a, the entropy curves are presented in Figure 11b–d when m=3,4,5 and τ=1. The detection performance of CPE algorithm for BF signal are basically consistent with those for IRF signal when m=3 and m=4. The detection difference of CPE for IRF and BF signals is that the result of the latter is obvious when m=5 according to Figure 10d and Figure 11d. As shown in Table 8, the Std at different *m* can also illustrate the above two aspects.

**Figure 12:**
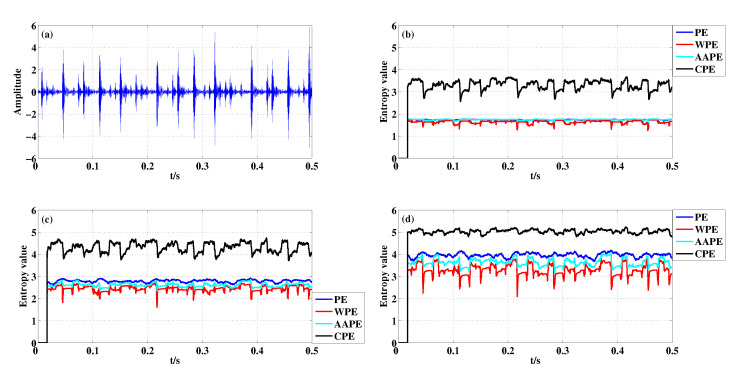
Detecting abnormal pulse in ORF signal with different *m* using PE, WPE, AAPE, and CPE: (**a**) The ORF signal; (**b**) m=3,τ=1; (**c**) m=4,τ=1; (**d**) m=5,τ=1.

Figure 12 presents the amplitude diagram of ORF signal and corresponding entropy curves of four kinds of algorithms at different *m*. When a fault pulse occurs, the entropy curve of CPE has a slight drop and then a low entropy of one window length appears. The reason is that the pulse intensity of ORF signal is higher than that of IRF signal. So, this different performance of CPE algorithm for IRF signal and ORF signal can help scholars distinguish these two faults better than WPE. Moreover, the four algorithms have basically the same performance for the pulse detection of IRF signal and ORF signal under the same parameters. Whether from Figure 12 or Table 9, the detection performance of CPE is optimal when m=3,4, and WPE seems to perform better when m=5.

Compared with the detection results of simulation data in Section 3, CPE does not seem to improve the detection performance much more than the other three algorithms for the actual data when m=5. In theory, for the same embedding dimension *m*, the larger the value of window length *w*, the larger the repetition rate of each permutation pattern in all position sequence, and so the advantage of CPE in dynamical change detection will be prominent. To illustrate this point from the perspective of simulation analysis, we employ the BF signal when t=5−10s. In this experiment, *w* is equal to 500, 800 and 1000 respectively under the condition of m=5, and the corresponding Std are presented in Table 10. From Table 10, the Std of CPE is increasing with the increase of *w* when m=5, and so the result supports the above theory.

The value of *w* in three models of Section 3 is equal to 2000, but it is equal to 200 or 500 for the actual signal. Therefore, the probability of appearing the same permutation pattern in all position sequence for the three models is larger than those of the actual signals when m=5. Therefore, the detection performance of CPE for the dynamical changes in the three models is more prominent than those for the actual signal when m=5. It is obvious that CPE has a better performance for dynamical change detection compared with the other three algorithms when there is a larger repetition rate of permutation pattern in position sequences.

## 5. Conclusions

In this paper, an improved algorithm named CPE is proposed to solve the problems existing in PE by employing the secondary partitioning. A synthetic signal, a discrete standard model and a continuous standard model are applied to verify the limitations of PE, WPE and AAPE, and illustrate the advantage of CPE. The dynamical change detection performance of these four algorithms are also studied when the signal contains the noise, and then the algorithm complexity is analyzed from the running time and time complexity. Finally, these four algorithms are used to analyze the detection performance of the actual signals. Simulation results show that CPE can amplify the results of dynamical change detection compared to PE, WPE and AAPE, whether for the synthetic signal, logistic map or Lorenz map. In particularly, CPE shows extremely accurate feature fetching for the logistic map and the Lorenz map when m=3 or m=4. However, CPE has less robustness to noise and requires a higher computation cost than PE, WPE and AAPE. The application of CPE in actual signals illustrate that CPE performs better than the other three algorithms in detecting abnormal pulse of rolling bearing when *m* is small. From all the analyses in this paper, we find that CPE has a better performance for dynamical change detection compared with the other three algorithms when there is a larger repetition rate of permutation pattern in position sequences. CPE is an extremely useful abnormal detection method, and may have more practical applications.

## Figures and Tables

**Table 1:** The jumps (Jump1, Jump2), and the Std of PE, WPE, AAPE, and CPE for the synthetic signal in different *m*. Jump1 is equal to Mv2 minus Mv1 and Jump2 is equal to Mv2 minus Mv3, where Mv1, Mv2 and Mv3 represent the mean of entropy value when t= 2 s–10 s, t= 12 s–20 s and t= 22 s–30 s respectively.

Synthetic Signal	m=3			m=4			m=5			m=6		
	Jump1	Jump2	Std	Jump1	Jump2	Std	Jump1	Jump2	Std	Jump1	Jump2	Std
PE	0.081	0.001	0.037	0.169	0.008	0.075	0.256	0.015	0.113	0.354	0.029	0.155
WPE	−0.006	0.006	0.005	0.000	0.007	0.003	0.014	0.009	0.006	0.037	0.012	0.015
AAPE	0.073	0.003	0.032	0.135	0.006	0.060	0.190	0.007	0.085	0.251	0.007	0.112
CPE	0.130	0.092	0.053	0.268	0.179	0.108	0.334	0.144	0.133	0.413	0.179	0.164

**Table 2:** The jumps when r=3.702 (Jump1) and r=3.961 (Jump2), and the Std of PE, WPE, AAPE, and CPE for the logistic map in different *m*. Jump1 is equal to the entropy value when r=3.701 minus those when r=3.702, and Jump2 is equal to the entropy value when r=3.96 minus those when r=3.961.

Logistic Map	m=3			m=4			m=5			m=6		
	Jump1	Jump2	Std	Jump1	Jump2	Std	Jump1	Jump2	Std	Jump1	Jump2	Std
PE	0.137	0.445	0.107	0.218	0.952	0.351	0.611	0.877	0.478	0.764	1.524	0.737
WPE	0.144	0.240	0.089	0.245	0.804	0.327	0.689	0.710	0.447	0.846	1.330	0.699
AAPE	0.132	0.375	0.098	0.219	0.937	0.356	0.655	0.850	0.551	0.821	1.503	0.801
CPE	0.397	0.857	0.385	0.589	1.467	0.657	1.003	2.091	0.844	1.223	2.750	1.106

**Table 3:** The jumps when r(t)=166 (Jump1) and r(t)=230.4 (Jump2), and the Std of PE, WPE, AAPE, and CPE for the Lorenz map in different *m*. Jump1 is equal to the entropy value when r(t)=166.4 minus those when r(t)=166, and Jump2 is equal to the entropy value when r(t)=227 minus those when r(t)=230.4.

Lorenz Map	m=3			m=4			m=5			m=6		
	Jump1	Jump2	Std	Jump1	Jump2	Std	Jump1	Jump2	Std	Jump1	Jump2	Std
PE	−0.073	−0.008	0.078	0.055	−0.003	0.154	0.355	0.038	0.375	0.953	0.181	0.587
WPE	0.013	0.016	0.120	0.143	0.006	0.226	0.442	0.041	0.374	0.992	0.202	0.570
AAPE	−0.033	0.008	0.097	0.117	0.012	0.152	0.390	0.043	0.376	0.959	0.195	0.579
CPE	0.299	0.033	0.246	0.809	0.414	0.479	1.286	0.660	0.693	1.605	0.852	0.880

**Table 4:** The running time of PE, WPE, AAPE, and CPE for the above three models in different *m*.

		Running Time (Second)			
		m=3	m=4	m=5	m=6
Synthetic signal	PE	1037	1668	3677	17534
	WPE	2293	2800	5315	19823
	AAPE	1390	1945	4239	17950
	CPE	1925	3021	5534	20811
Logistic map	PE	8	16	53	290
	WPE	29	36	73	309
	AAPE	15	24	71	386
	CPE	21	37	89	365
Lorenz map	PE	26	41	134	711
	WPE	81	100	183	765
	AAPE	40	57	151	712
	CPE	74	162	441	1367

**Table 5:** The Gr of running time of CPE referring to PE in different *m*.

	Gr			
	m=3	m=4	m=5	m=6
Synthetic signal	86%	81%	51%	19%
Logistic map	163%	131%	68%	26%
Lorenz map	185%	295%	229%	92%
Average	145%	169%	116%	55.5%

**Table 6:** The time complexity of PE, WPE, AAPE, and CPE algorithms.

Algorithm	Time Complexity
PE	$O\left((N-(m-1)\tau )\cdot m!\right)$
WPE	$O\left((N-(m-1)\tau )\cdot 4m!\right)$
AAPE	$O\left((N-(m-1)\tau )\cdot m!(m-1)\right)$
CPE	$O\left((N-(m-1)\tau )\cdot m!(m+1)\right)$

**Table 7:** The Std of PE, WPE, AAPE, and CPE for IRF signal in different *m*.

IRF	Std		
	m=3	m=4	m=5
PE	0.010	0.061	0.096
WPE	0.043	0.122	0.261
AAPE	0.011	0.068	0.127
CPE	0.165	0.176	0.081

**Table 8:** The Std of PE, WPE, AAPE, and CPE for BF signal in different *m*.

BF	Std		
	m=3	m=4	m=5
PE	0.004	0.048	0.094
WPE	0.015	0.053	0.113
AAPE	0.004	0.048	0.100
CPE	0.061	0.109	0.097

**Table 9:** The Std of PE, WPE, AAPE, and CPE for ORF signal in different *m*.

ORF	Std		
	m=3	m=4	m=5
PE	0.012	0.059	0.093
WPE	0.062	0.120	0.251
AAPE	0.020	0.083	0.167
CPE	0.214	0.227	0.088

**Table 10:** The Std of the entropy value for the BF signal when changing the value of *N* under the condition of m=5.

	Std			
	PE	WPE	AAPE	CPE
*w* = 500	0.0904	0.1179	0.1010	0.1047
*w* = 800	0.0701	0.0995	0.0821	0.1186
*w* = 1000	0.0629	0.0940	0.0758	0.1218

## Abbreviations

The following abbreviations are used in this manuscript:

PE	Permutation Entropy
WPE	Weighted Permutation Entropy
AAPE	Amplitude-aware Permutation Entropy
MvPE	Multivariate Permutation Entropy
CPE	Coded Permutation Entropy
Std	Standard Deviation
SNR	Signal-to-Noise Ratio
Gr	Growth Rate
IRF	Inner Race Fault
BF	Ball Fault
ORF	Outer Race Fault
